# Population Dynamics of *Listeria monocytogenes* and Yeast and Mold Levels on Different Pear Varieties During Simulated Storage

**DOI:** 10.3390/foods14101701

**Published:** 2025-05-11

**Authors:** Mengqian Hang, Edmund Larbi Afari, Xiaoye Shen, Yuan Su, Manoella Mendoza, Ines Hanrahan, Mei-Jun Zhu

**Affiliations:** 1 School of Food Science, Washington State University, Pullman, WA 99164, USA; mengqian.hang@wsu.edu (M.H.); edmund.afari@wsu.edu (E.L.A.); xiaoye.shen@wsu.edu (X.S.); yuan.su@wsu.edu (Y.S.); 2 Tree Fruit Research Commission, Wenatchee, WA 98801, USA; manoella@treefruitresearch.com (M.M.); hanrahan@treefruitresearch.com (I.H.)

**Keywords:** *Listeria monocytogenes*, pears, varieties, cold storage, temperature, cropping season, yeast and mold

## Abstract

This study examined the attachment, persistence, and fate of *Listeria monocytogenes* on selected varieties of fresh pears during simulated extended storage, along with the dynamics of resident yeast and mold (Y&M) populations. Following inoculation with ~7.0 log CFU/mL, *L. monocytogenes* exhibited varying attachment efficiencies across pear varieties, resulting in 6.23 ± 0.03, 6.30 ± 0.01, and 5.12 ± 0.01 log CFU/pear on Bartlett, d’Anjou, and Bosc pears, respectively, after 24 h. The *L. monocytogenes* population gradually declined on pear surfaces during storage. After 14 days, *L. monocytogenes* populations decreased to ~4.20, 5.96, and 4.07 log CFU/pear on Bartlett, d’Anjou, and Bosc pears, respectively, regardless of temperature, and remained stable over the subsequent 14 days. During 20-week storage at 0 °C, the *L. monocytogenes* level decreased by 2.65–3.84 log CFU/pear on all pear varieties. Y&M levels varied across pear varieties and crop years. The initial Y&M counts for Bartlett, d’Anjou, and Bosc in year 2 were 4.37 ± 0.01, 5.93 ± 0.02, and 5.11 ± 0.03 log CFU/pear, respectively. The Y&M levels of d’Anjou and Bosc for years 1 and 2 were 4.73–4.79 and 5.11–5.93 log CFU/pear, respectively. During 20-week storage at 0 °C, Y&M counts generally increased, with Bartlett pears exhibiting a more pronounced rise after 12 weeks. Data indicated that *L. monocytogenes* did not grow on pears; instead, its population declined under all simulated storage conditions, offering practical guidance for pear packers on *Listeria* behaviors under various storage conditions.

## 1. Introduction

*Listeria monocytogenes* is a major foodborne pathogen, resulting in ~1600 illnesses and ~260 deaths annually in the U.S. [1,2]. The dominant food vehicles of *L. monocytogenes* outbreaks have shifted over time, with fresh produce being the leading category in 2018–2023, accounting for most listeriosis cases [3]. The 2014–2015 listeriosis outbreak linked to caramel apples, which caused 35 illnesses and 7 deaths [4], along with multiple recalls of fresh apples due to potential contamination [5,6,7,8,9], highlights the importance of controlling *L. monocytogenes* on pome fruits. Once contaminated, *L. monocytogenes* could survive and even grow on fruit surfaces during extended refrigeration due to its psychotropic nature [10,11,12,13]. This poses a significant concern for fresh apples and pear packers, as these fruits are typically stored under 0–2 °C for 6–12 months before packing [14,15,16]

The survival of *L. monocytogenes* on apples has been studied across various cultivars and storage conditions, with the pathogen persisting throughout storage [11,12,17,18,19,20,21]. However, limited data exist on *L. monocytogenes* behavior on pears during storage. Pear, a popular pome fruit, is widely consumed for its low caloric value and high nutritional content [22]. In 2021, U.S. fresh pear consumption reached 562,470 tons [23], with Washington State being the largest producer, yielding 285,000 tons in 2022 [24]. Differences in postharvest handling [15,25] and fruit surface characteristics, such as skin and epicuticular wax composition [26,27], may influence *L. monocytogenes* survival dynamics on pears compared to apples.

Additionally, postharvest decay leads to substantial economic losses of pome fruits, from 5 to 20%, and up to 50% on susceptible cultivars [28]. In South Africa, pear decay accounts for 14% of total gross fruit losses [29]. This study aims to evaluate the fate of *L. monocytogenes* and resident yeast and mold (Y&M) levels on pear surfaces during storage, considering three important pear varieties and storage temperatures.

## 2. Materials and Methods

### 2.1. L. monocytogenes Strains and Culture Preparation

*L. monocytogenes* strains NRRL B-57618 (serotype ½ a), NRRL B-33053 (serotype 4b), and NRRL B-33466 (serotype ½ b) were obtained from the USDA-ARS culture collection (National Center for Agricultural Utilization Research (NRRL), Peoria, IL, USA). The cultures were preserved in Trypticase Soy Broth (Fisher Scientific, Fair Lawn, NJ, USA), supplemented with 0.6% yeast extract (*w*/*v*) (TSBYE, Fisher Scientific) and 20% (*v*/*v*) glycerol at −80 °C. The frozen culture, prepared based on a method used by [30], was activated in TSBYE for 24 h at 37 °C, followed by sub-culturing in TSBYE for another 24 h at 37 °C. The cultures were washed in sterile 1× phosphate-buffered saline (PBS, pH 7.4) once, then an equal volume of three strains was combined to obtain a three-strain cocktail for pear inoculation.

### 2.2. Pear Inoculation

Whole, fresh, and unwaxed conventional Bartlett, d’Anjou, or Bosc pears at commercial maturity without bruises or cuts were washed with tap water and dried at room temperature (RT, 22 ± 1 °C) before inoculation. The pears were then submerged in the prepared *L. monocytogenes* inoculum for 2 min followed by drying at RT for 24 h to allow for bacteria to establish. Sets of 10–12 pears were randomly sampled at 0 and 24 h post-inoculation to confirm the inoculum uniformity, attachment, and initial *L. monocytogenes* levels on pear surfaces.

### 2.3. Storage Treatment of Inoculated Pears

The 24 h post-inoculated pears were randomly aligned on fiber fruit trays, with each tray holding pears from all inoculation batches, and stored at 0 °C, 10 °C, or 22 °C, representing the typical commercial storage temperature, temperature abuse setting, and RT, respectively [17,25]. The temperatures were monitored and maintained at 10.12 ± 0.24 °C for the 10 °C treatment and 0.60 ± 0.14 °C for the 0 °C storage. Sets of 10–12 pears were sampled at selected time points from each temperature treatment to enumerate *L. monocytogenes* and resident Y&M levels. Sampling was performed following the previously described method [12] with slight modifications. For short-term storage, inoculated pears were sampled on days 1, 4, 7, 14, and 28. For long-term storage, sampling occurred at weeks 1, 2, 4, 8, 12, 16, and 20 during each of two consecutive years. Each year was treated as an independent replicate, and data from the two years were analyzed separately.

### 2.4. Microbiological Analysis

Each pear was transferred to a sterile stomacher bag containing 10 mL PBS with 0.1% sodium thiosulfate (ST, Fisher Scientific) and 0.01% Tween 80 (Sigma-Aldrich, St. Louis, MO, USA), followed by hand-rubbing for 80 s. After rubbing, the stem and calyx areas were removed using a sterile knife and transferred to the rubbing solution, then homogenized for 2 min at 260 rpm using a Seward stomacher 400 circulator (Worthing, West Sussex, UK). To evaluate an alternative microbial detachment method, a peeling enumeration approach was compared with the hand rubbing–stomaching method. Briefly, unrubbed pears were peeled using a stainless-steel vegetable peeler, with the stem and calyx areas removed following the same procedure as described above. The peels, along with the stem and calyx areas, were transferred to a sterile stomacher bag containing 10 mL PBS with 0.1% ST and 0.01% Tween 80, then homogenized under the same conditions.

The homogenized solutions were serially diluted 10-fold in PBS, and the appropriate dilutions were plated onto TSAYE (TSBYE with 1.5% agar) and Potato Dextrose Agar (PDA) with chloramphenicol plates for *L. monocytogenes* and Y&M enumeration, respectively. The TSAYE plates were incubated at 37 °C for 24 h, and the PDA plates were incubated at RT for 5 days.

### 2.5. Statistical Analysis

Data were analyzed using one-way Analysis of Variance (ANOVA), followed by Tukey’s multiple comparison test, using IBM SPSS Statistics software (Version 26, Chicago, IL, USA). Graphs were generated using GraphPad Prism version 9.0 (GraphPad Software, Boston, MA, USA). A *p*-value of <0.05 was considered statistically significant.

## 3. Results

### 3.1. Attachment Behavior of L. monocytogenes on Pear Surfaces

**Figure 1:**
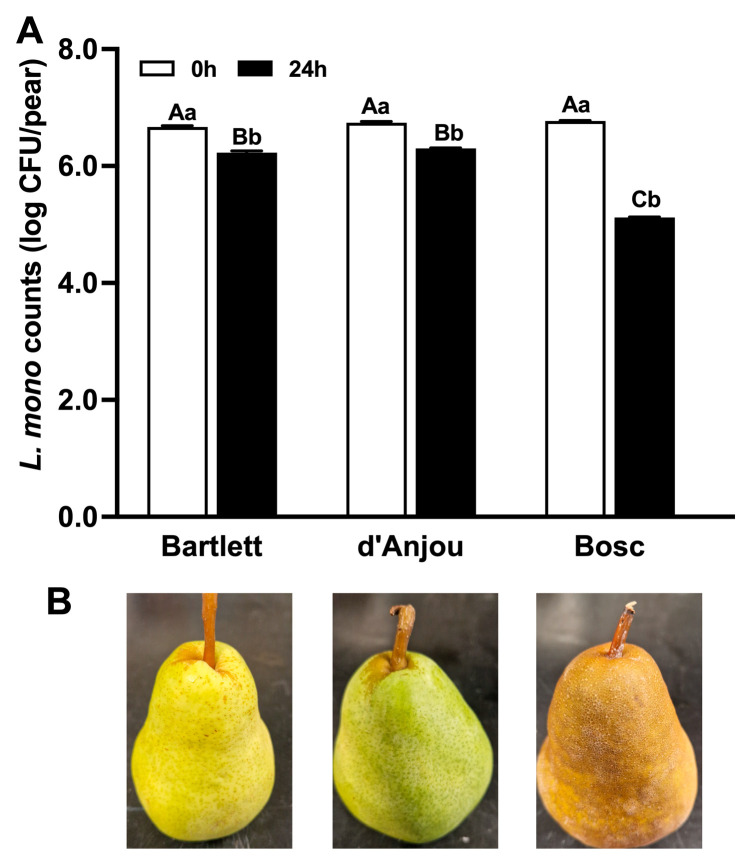
Attachment behavior of *L. monocytogenes* on different pear varieties. (**A**) *L. monocytogenes* populations at 0 and 24 h post-inoculation. (**B**) Representative images of Bartlett, d’Anjou, and Bosc pears. Letters A–C above bars indicate significant differences in *L. monocytogenes* levels among different pear varieties at each sampling point (*p* < 0.05). Different letters (a, b) within same variety indicate significant differences over time (*p* < 0.05). Mean ± SEM, *n* = 30.

**Figure 2:**
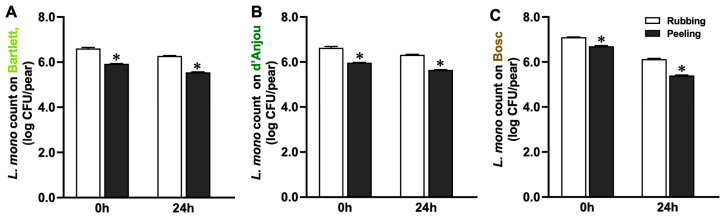
Detachment of *L. monocytogenes* from fresh Bartlett (**A**), d’ Anjou (**B**), and Bosc (**C**) pears using rubbing and peeling methods. Mean ± SEM, *n* = 30. * Indicates a significant difference (*p* < 0.05) between peeling and rubbing methods at 0 and 24 h post-inoculation.

Immediately after inoculation, the initial levels of *L. monocytogenes* on pears were 6.67–6.77 log CFU/pear, regardless of variety (Figure 1A). After 24 h of establishment at RT, *L. monocytogenes* levels on pear surfaces decreased (*p* < 0.05), particularly on Bosc pears, resulting in levels of 6.23 ± 0.03, 6.30 ± 0.01, and 5.12 ± 0.01 log CFU/pear on the Bartlett, d’Anjou, and Bosc pears, respectively (Figure 1A). The visual appearances of the three pear varieties post-inoculation are displayed in Figure 1B, showcasing the morphological difference between the varieties. The peeling detachment method resulted in *L. monocytogenes* levels that were 60–80% lower (*p* < 0.05) compared to the rubbing method, regardless of pear variety (Figure 2). Consequently, the rubbing method was selected for subsequent pear storage studies.

### 3.2. L. monocytogenes Survival on Pears Under Different Storage Temperatures

**Figure 3:**
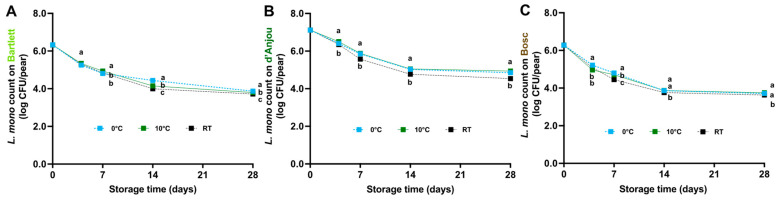
Survival of *L. monocytogenes* on Bartlett (**A**), d’Anjou (**B**), and Bosc (**C**) pears during 28 days of storage at 0 °C, 10 °C, and RT (~22 °C). Mean ± SEM, *n* = 12. Different letters (a–c) indicate significant differences between temperatures at the same sampling point (*p* < 0.05).

*L. monocytogenes* populations gradually decreased on pears over 28 days of storage, regardless of the storage temperature, with the most significant reductions occurring in the first 14 days (Figure 3). After 14 days of storage at 0 and 10 °C, the populations of *L. monocytogenes* decreased (*p* < 0.05) to 4.30 ± 0.03, 5.04 ± 0.02, and 3.87 ± 0.02 log CFU/pear on the Bartlett, d’Anjou, and Bosc pears, respectively. During the remaining 14 days of storage, the populations remained relatively stable at 3.75–4.89 log CFU/pear (Figure 3). Storing the pears at RT resulted in a slightly, but significantly greater reduction compared to the other storage temperatures. The population of *L. monocytogenes* was maintained at 3.63–4.54 log CFU/pear by the end of 28-day storage at RT, regardless of pear variety (Figure 3).

**Figure 4:**
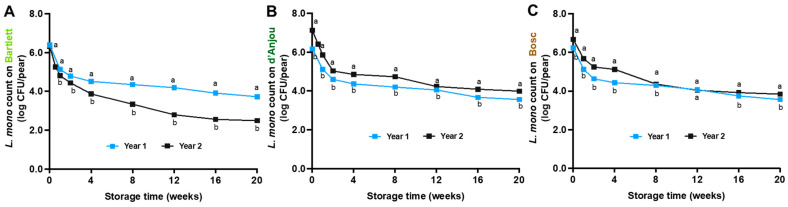
Survival of *L. monocytogenes* on fresh Bartlett (**A**), d’Anjou (**B**), and Bosc (**C**) pears during 20 weeks of storage at 0 °C across two crop years. Mean ± SEM, *n* = 12. Different letters (a, b) indicate significant differences between years at the same sampling point (*p* < 0.05).

*L. monocytogenes* gradually decreased on pears during long-term storage at 0 °C, exhibiting similar survival patterns across different pear varieties. After 20 weeks of storage at 0 °C, the *L. monocytogenes* population decreased by 2.65 ± 0.03, 2.86 ± 0.03, and 2.83 ± 0.02 log CFU/pear on Bartlett, d’Anjou, and Bosc pears, respectively, in year 1, and 3.84 ± 0.03, 3.13 ± 0.03, and 2.82 ± 0.03 log CFU/pear, respectively, in year 2 (Figure 4).

### 3.3. Fate of Yeasts and Molds on Pears During Storage

**Figure 5:**
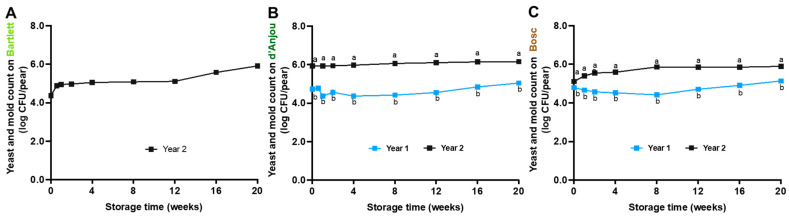
Survival of yeast and mold on Bartlett (**A**), d’Anjou (**B**), and Bosc (**C**) pears during 20 weeks of storage at 0 °C. Mean ± SEM, *n* = 12. Different letters (a, b) indicate significant differences between years at the same sampling point (*p* < 0.05).

The initial Y&M levels on d’Anjou pears varied between cropping years and pear varieties (*p* < 0.05). In year 1, Y&M levels were 4.8–4.9 log CFU/pear on d’Anjou and Bosc pears. In year 2, the counts were 4.37 ± 0.01, 5.93 ± 0.02, and 5.11 ± 0.03 log CFU/pear for Bartlett, d’Anjou, and Bosc pears, respectively. During 20 weeks of storage at 0 °C, Y&M counts generally increased on the Bartlett (year 2), d’Anjou (year 1), and Bosc pears (years 1 and 2), while remaining relatively stable at ~6.0 log CFU/pear on the d’Anjou pears in year 2 (Figure 5).

## 4. Discussion

### 4.1. Attachment Behavior of L. monocytogenes on Pears

The observed decline in *L. monocytogenes* populations on pear surfaces after the attachment period aligns with the previous findings in other fruits. The *L. monocytogenes* population decreased by 0.6 log CFU/peach on fresh peaches 24 h after dip-inoculation at RT [31]. The population of *L. monocytogenes* serotype 4b strains decreased by 1.1–1.4 log CFU/apple on inoculated apples during 48 h of holding at RT [21]. However, *L. monocytogenes* 1/2a and 1/2b strains were reported to increase on apples 24–48 h after inoculation [21]. These variations may be due to differences in bacteria serotypes and strains, fruit types, and inoculation environments. Fruit surface characteristics, such as epicuticular wax composition and micro-structures, could influence bacterial adhesion [32,33]. In the current study, the attachment of *L. monocytogenes* varied among pear varieties, likely due to cultivar-specific ecological factors, such as fruit surface characteristics, surface nutrients, epicuticular wax compositions, and micro-gas environments [34,35]. Kovacs et al. [36] demonstrated that different pear varieties exhibited unique structural differences, which could influence microbial persistence. Additionally, Amarante et al. [37] highlighted that Bartlett pears, with their non-lignified smooth skins, have different permeabilities to water vapor and gases compared to Bosc pears, which have a rough skin. Pear surfaces generally contain low levels of nutrients, which may help defend against microbial infestation [38]. Moreover, the decrease in *L. monocytogenes* populations on the pear surface could represent them adapting to a potential viable but non-culturable (VBNC) state [39]. The method used for detaching microorganisms also impacts bacterial recovery from fruit surfaces [40]. This study confirmed that meticulous hand massaging followed by stem–calyx stomaching was more effective in detaching microorganisms from pear surfaces, resulting in significantly higher bacterial recovery compared to the peeling method. Therefore, the rubbing method was used in subsequent studies to maximize microbial recovery from pear surfaces. Using the peeling method would likely have resulted in lower recovery and the underreporting of *L. monocytogenes* levels.

### 4.2. Fate of L. monocytogenes on Pears During Storage

The *L. monocytogenes* population gradually decreased on pear surfaces during storage, irrespective of pear variety and temperature. This trend aligns with previous findings on apples, where *L. monocytogenes* populations decreased by 1.1–2.0 log CFU/apple over 2–6 weeks at RT or over 12 weeks at 1 °C [12,20]. Similarly, a reduction of 2.9–4.4 log CFU/apple of *L. monocytogenes* was reported on HoneyCrisp and Fuji apples after 60 days at 5 °C, 35 days at 12 °C, or 7 days at 22.5 °C [17]. In the present study, 20-week storage at 0 °C led to a ~3.0 log CFU/pear reduction in *L. monocytogenes*. Comparable reductions (2.1–2.9 log CFU/apple) were observed for *Listeria innocua* on Granny Smith and Red Delicious apples stored in commercial Refrigerated air (RA) conditions at 0.2–0.6 °C for 36 weeks [16,30]. Likewise, a similar decline in *L. monocytogenes* was reported across various types of whole produce, including apples, cantaloupes, and mangoes, where significant reductions were attributed to the limited availability of accessible nutrients on the produce surfaces [41]. In contrast, *L. monocytogenes* increased by 0.34 log CFU/g on fresh-cut pears stored at 4 °C for 4 days [42], by 0.18 log CFU/g on fresh cut-pears stored at 1 °C for 9 days [43], and by 2.05 log CFU/g in fresh-cut pears after 7 days of storage at 10 °C [44]. This growth likely resulted from the available nutrients and conducive conditions on fresh-cut pears [45].

Elevated storage temperature (RT) led to a slight but significant increase in the *L. monocytogenes* reduction on pear surfaces. Similarly, greater reductions were observed on Fuji and HoneyCrisp apples stored at 12 and 22.5 °C compared to 5 °C [17]. However, *L. monocytogenes* remained detectable after 20 weeks, underscoring the need for additional antimicrobial interventions. Treatments such as gaseous ozone [13,16,30,46] and chlorine dioxide [47] can help further mitigate contamination risks during pear storage. Proper handling throughout production and at the postharvest, distribution, retail, and consumer stages is also essential to ensure microbial safety along the supply chain.

Despite its relevance, this study has limitations. Pears were inoculated with high *L. monocytogenes* levels to ensure detectable reductions, which may not represent natural contamination. Additionally, the simulated storage conditions may not fully reflect commercial practices, warranting further investigation.

### 4.3. Levels of Resident Yeast and Mold During Pear Storage

Pears are prone to postharvest decay such as blue mold, gray mold, and mucor rot [48,49,50]. Understanding the dynamics of spoilage microbiota during storage is critical to reducing postharvest losses in pears. In this study, the resident Y&M population on pear surfaces exhibited a slight increase during storage, with significant variations observed among pear varieties and crop years. Similarly, the Y&M counts on fresh Fuji and Red Delicious apples increased by 1.0–1.3 log CFU/apple during 36 weeks of RA storage at 0.2–0.6 °C [13,16]. The Y&M counts on Granny Smith apples harvested in two consecutive crop years ranged from 4.6 to 5.0 log CFU/apple, which slightly increased by 0.4–0.7 log CFU/apple during 30–36 weeks of storage at 0.6 °C [30]. Additionally, the Y&M population on carrots increased by 1.5 log CFU/g after 7 days of storage at 15 °C [51]. Fungal populations on fruits are impacted by factors such as climatic conditions during the crop year, soil properties, and harvest timing [52,53].

The differences in Y&M levels among the pear varieties suggest that variety-specific characteristics, such as skin properties, and intrinsic characteristics may influence fungal dynamics. Hou et al. [54] demonstrated that intrinsic properties like titratable acidity and soluble solid content can influence the fungal diversity during storage. Similarly, Zhang et al. [55] reported that the growth of *Alternaria alternata* was affected by the pear’s surface hydrophobicity, suggesting that differences in cuticular wax composition among pears may influence Y&M counts. Gao et al. [56] further demonstrated that specific characteristics of pears can shape fungal growth patterns. Furthermore, the variation in Y&M levels across crop years may be influenced by differences in environmental conditions during cultivation. Factors such as temperature, humidity, and rainfall can impact microbial communities on pear surfaces throughout the growing season, potentially contributing to interannual variability [57]. Fungal levels on fruit surfaces can serve as indicators of postharvest disease risk. These findings highlight the intricate relationship between pear varieties, intrinsic characteristics, and environmental factors in shaping Y&M dynamics on pears.

## 5. Conclusions

The *L. monocytogenes* population gradually declined on pear surfaces over time, yet residual cells persisted after 20 weeks of storage. Attachment and persistence varied by pear varieties and crop year. Resident Y&M counts slightly increased during storage, with significant variations depending on variety and season. These findings suggest that while *L. monocytogenes* does not grow on pears, it can persist, providing practical insights for packers on its behaviors during storage. To further reduce contamination risks and postharvest losses, incorporating antimicrobial interventions such as ozone during extended storage may offer added protection.

## Data Availability

The original contributions presented in this study are included in the article; further inquiries can be directed to the corresponding author.
